# Variability in Temperature-Related Mortality Projections under Climate Change

**DOI:** 10.1289/ehp.1306954

**Published:** 2014-07-18

**Authors:** Tarik Benmarhnia, Marie-France Sottile, Céline Plante, Allan Brand, Barbara Casati, Michel Fournier, Audrey Smargiassi

**Affiliations:** 1 Département de santé environnementale et santé au travail (DSEST), Université de Montréal, Montréal, Québec, Canada; 2 EHESP School of Public Health (École des hautes études en santé publique), Rennes, Sorbonne-Paris Cité, Rennes, France; 3 Chaire sur la pollution de l’air, les changements climatiques et la santé, DSEST, Université de Montréal, Montréal, Québec, Canada; 4 Consortium Ouranos, Montréal, Québec, Canada; 5 Ministère du Développement durable, de l’Environnement et des Parcs, Québec, Canada; 6 Direction de santé publique de l’Agence de la santé et des services sociaux de Montréal, Québec, Canada; 7 Institut National de Santé Publique du Québec, Montréal, Québec, Canada

## Abstract

Background: Most studies that have assessed impacts on mortality of future temperature increases have relied on a small number of simulations and have not addressed the variability and sources of uncertainty in their mortality projections.

Objectives: We assessed the variability of temperature projections and dependent future mortality distributions, using a large panel of temperature simulations based on different climate models and emission scenarios.

Methods: We used historical data from 1990 through 2007 for Montreal, Quebec, Canada, and Poisson regression models to estimate relative risks (RR) for daily nonaccidental mortality in association with three different daily temperature metrics (mean, minimum, and maximum temperature) during June through August. To estimate future numbers of deaths attributable to ambient temperatures and the uncertainty of the estimates, we used 32 different simulations of daily temperatures for June–August 2020–2037 derived from three global climate models (GCMs) and a Canadian regional climate model with three sets of RRs (one based on the observed historical data, and two on bootstrap samples that generated the 95% CI of the attributable number (AN) of deaths). We then used analysis of covariance to evaluate the influence of the simulation, the projected year, and the sets of RRs used to derive the attributable numbers of deaths.

Results: We found that < 1% of the variability in the distributions of simulated temperature for June–August of 2020–2037 was explained by differences among the simulations. Estimated ANs for 2020–2037 ranged from 34 to 174 per summer (i.e., June–August). Most of the variability in mortality projections (38%) was related to the temperature–mortality RR used to estimate the ANs.

Conclusions: The choice of the RR estimate for the association between temperature and mortality may be important to reduce uncertainty in mortality projections.

Citation: Benmarhnia T, Sottile MF, Plante C, Brand A, Casati B, Fournier M, Smargiassi A. 2014. Variability in temperature-related mortality projections under climate change. Environ Health Perspect 122:1293–1298; http://dx.doi.org/10.1289/ehp.1306954

## Introduction

Among environmental determinants of health, weather and climate have received increasing attention related to awareness of climate change and the documentation of both usual and catastrophic heat-related mortality (Campbell-Lendrum and Woodruff 2007; Ebi 2008; Kovats and Hajat 2008; O’Neill and Ebi 2009; Patz et al. 2005).

Increases in ambient summer temperatures over city-specific thresholds have been associated with an increase in mortality [Hajat and Kosatsky 2010; Intergovernmental Panel on Climate Change (IPCC) 2007a, 2007b]. Although present-day health effects of summer temperatures have been well characterized (Armstrong 2006), the extent to which future changes in summer temperatures will affect human health has received relatively little attention (Campbell-Lendrum and Woodruff 2007; Costello et al. 2009; Ebi and Gamble 2005; Huang et al. 2011; McMichael et al. 2006; Peng et al. 2011). Global average ambient temperatures have been projected to increase under any scenario of increasing greenhouse gas (GHG) concentrations (IPCC 2001, 2007a). Various models have been developed in climate science (Caya et al. 1995; McFarlane et al. 1992) to estimate future temperatures according to different GHG emission scenarios [IPCC *Special Report on Emissions Scenarios* (SRES); IPCC 2000].

Studies published since 2008 that have estimated the impacts of future temperatures on mortality have mainly been conducted in Europe and North America, and the time periods used for baseline data and projections have varied among them. Most studies have evaluated projected temperatures based on only a small number of climate change simulations, with the exception of Li et al. (2013). For a review of most of these studies, see Huang et al. (2011) and Gosling et al. (2009).

Divergences in temperature projections, for example, due to model structure and GHG emissions scenarios, may occur; to capture the maximal range of possible future temperatures and health impacts, it may thus be important to consider a large number of simulations when assessing health impacts.

We assessed the variability of temperature projections and future mortality distributions, using a large panel of temperature simulations based on climate models and emission scenarios for the period 2020–2037 on the island of Montreal, which includes the city of Montreal, the largest city of the province of Quebec, Canada.

## Methods

To predict mortality attributable to past (1990–2007) and future temperatures (2020–2037) in Montreal, we first used historical data for 1990–2007 to estimate three sets of relative risks (RRs) for associations between mortality and temperatures during June–August on the island of Montreal with Poisson models. We refer to “sets” of RRs, given that there is one RR per degree temperature due to the nonlinear relation between temperature and mortality. One set of RRs was based on the observed historical data, and two were based on individual bootstrap samples constructed from the observed historical data that generated the 95% confidence interval (CI) of the attributable number (AN) of deaths. We then used the three sets of RRs to predict mortality attributable to past (1990–2007) and future temperatures (2020–2037), using the observed historical temperatures and 32 different temperature simulations (for 1990–2007 and 2020–2037) based on three general circulation models (GCMs) and the Canadian Regional Climate Model (RCM) (Caya et al. 1995). Predicted temperature distributions were corrected by applying the daily translation method (Mpelasoka and Chiew 2009) based on observed versus simulated temperatures for 1990–2007. Finally, using analysis of covariance (ANCOVA), we examined factors that influence the variability in future ANs, including the three sets of RRs and the 32 temperature simulations used to estimate future ANs (2020–2037). The project was carried out in the context of the Quebec ministerial health surveillance plan, which obtained ethics approval from the Quebec Public Health Ethical Health Surveillance Committee.

*Observations*. Mortality data. The mortality file comprises data on residents of Montreal who died on the island during June, July, and August of 1990 through 2007. We included all underlying nonaccidental causes of death and excluded deaths for *International Classification of Diseases, Revision 9* (ICD-9) codes 800–999 (injury and poisoning) and *Revision 10* (ICD-10) codes S00-T98 (injury, poisoning, and other consequences of external causes).

Observed temperatures and ozone (O_3_) levels. We computed daily (0000–2300 hours) mean, minimum, and maximum outdoor temperatures using data for June–August 1990–2007 obtained from the Environment Canada meteorological observation station at the Montréal Pierre Elliott Trudeau International Airport (Environment Canada 2012), located about 20 km from the city core (Doyon et al. 2008). We obtained hourly measurements of O_3_ at seven fixed-site monitoring stations from the Environment Canada National Air Pollution Surveillance Network (Environment Canada 2012). We averaged hourly concentrations over all stations and computed daily mean concentrations of O_3_ from these values for the summers (June–August) of 1990–2007.

*Projection of temperatures and methods applied to correct simulated temperatures*. Models and simulations. The 32 simulations used in the present study provided climate data for 1990–2007 and 2020–2037, as well as daily temperature estimates (Table 1). We selected the near future period 2020–2037 for projections to provide a climate change signal out of the climate natural variability that still corresponds to a near future for public health consideration. For each climate model, we considered the temperature time series for the grid point nearest to Montreal. Twenty-two of the simulations used in this study were based on the GCM simulations of the World Climate Research Programme (WCRP) Coupled Model Intercomparison Project, phase 3 (CMIP3) multimodel data set (Meehl et al. 2007), which used three SRES GHG emissions scenarios: B1, A1B, and A2 representing mild, medium, and strong future emissions of greenhouse gases and aerosols, respectively (IPCC 2000). GCMs have a resolution of 200–300 km, and the RCM has a resolution of approximately 50 km. The ensemble of the CMIP3 climate models is the multimodel data set used for the IPCC Fourth Assessment Report (IPCC 2007a). The CMIP3 simulations are available from the Program for Climate Model Diagnostic and Intercomparison archive (http://www-pcmdi.llnl.gov). Most of the CMIP3 GCMs covered three discrete time periods (1961–2000, 2046–2065, and 2081–2100), whereas the time periods selected for the present study (1990–2007 and 2020–2037) limited our choices to three GCMs with continuous simulations. Therefore, we used 15 simulations (with varying initial conditions) of the Canadian Global Circulation Model, version 3.1 (CGCM3.1/T47; Flato et al. 2000), developed at the Canadian Centre for Climate Modeling & Analysis (CCCMA) and three simulations of the same model truncated at a finer resolution (CGCM3.1/T63); three simulations of the Mark 3.5 (Mk3.5) climate model developed at the CSIRO Atmospheric Research, Australia (Cai et al. 2005); and one simulation of the German Coupled Global Climate Model (ECHAM5; Jungclaus et al. 2006) developed at the Max Planck Institute (MPI) of Meteorology (Germany). See Supplemental Material, Figure S1, for grids representing the GCM models.

**Table 1:** Simulations of temperatures and climate models used.

Simulation name	Climatic model	Pilot^*a*^	Member^*b*^	SRES	Domain	Temp variables
RCM
RCM1	MRCC 4.2.3	cccma_cgcm3_1	run5	sresA2	North America	All
RCM2	MRCC 4.2.3	cccma_cgcm3_1	run4	sresA2	North America	All
RCM3	MRCC 4.2.3	echam5	run1	sresA2	North America	All
RCM4	MRCC 4.1.1	cccma_cgcm3_1	run4	sresA2	Quebec	All
RCM5	MRCC 4.1.1	cccma_cgcm3_1	run5	sresA2	Quebec	All
RCM6	MRCC 4.2.3	cccma_cgcm3_1	run4	sresA2	Quebec	All
RCM7	MRCC 4.2.0	cccma_cgcm3_1	run4	sresA2	North America	All
RCM8	MRCC 4.2.0	cccma_cgcm3_1	run5	sresA2	North America	All
RCM9	MRCC 4.2.3	cccma_cgcm3_1	run5	sresA2	Quebec	All
RCM10	MRCC 4.2.3	echam5	run1	sresA2	Quebec	All
GCM
GCM1	cccma_cgcm3_1	NA	run1	sresa1b	NA	All
GCM2	cccma_cgcm3_1	NA	run1	sresa2	NA	All
GCM3	cccma_cgcm3_1	NA	run1	sresb1	NA	All
GCM4	cccma_cgcm3_1	NA	run2	sresa1b	NA	All
GCM5	cccma_cgcm3_1	NA	run2	sresa2	NA	All
GCM6	cccma_cgcm3_1	NA	run2	sresb1	NA	All
GCM7	cccma_cgcm3_1	NA	run3	sresa1b	NA	All
GCM8	cccma_cgcm3_1	NA	run3	sresa2	NA	All
GCM9	cccma_cgcm3_1	NA	run3	sresb1	NA	All
GCM10	cccma_cgcm3_1	NA	run4	sresa1b	NA	All
GCM11	cccma_cgcm3_1	NA	run4	sresa2	NA	All
GCM12	cccma_cgcm3_1	NA	run4	sresb1	NA	All
GCM13	cccma_cgcm3_1	NA	run5	sresa1b	NA	All
GCM14	cccma_cgcm3_1	NA	run5	sresa2	NA	All
GCM15	cccma_cgcm3_1	NA	run5	sresb1	NA	All
GCM16	cccma_cgcm3_1_t63	NA	run1	sresa1b	NA	Mean only
GCM17	cccma_cgcm3_1_t63	NA	run1	sresa2	NA	Mean only
GCM18	cccma_cgcm3_1_t63	NA	run1	sresb1	NA	Mean only
GCM19	csiro_mk3_5	NA	run1	sresa1b	NA	All
GCM20	csiro_mk3_5	NA	run1	sresa2	NA	All
GCM21	csiro_mk3_5	NA	run1	sresb1	NA	All
GCM22	mpi_echam5	NA	run4	sresa1b	NA	All
Abbreviations: cccma, Canadian Centre for Climate Modeling and Analysis; cgcm3_1, Canadian Global Circulation Model, version 3.1; csiro, Commonwealth Scientific and Industrial Research Organisation, Atmospheric Research; echam5, ECHAM5 (German Coupled Global Climate Model); mpi, Max Planck Institute; MRCC, Modèle régional canadien du climat (Canadian Regional Climate Model); NA, not applicable; sres, IPCC *Special Report on Emissions Scenarios*; Temp, temperature. ^***a***^GCM used to drive the RCM.^*** b***^Set of initial conditions.						

In addition to the GCM simulations, we used 10 simulations of the Canadian Regional Climate Model (CRCM; Caya et al. 1995; Laprise 2008; Plummer et al. 2006), versions 4.1 and 4.2 (de Elía and Côté 2010; Paquin 2010) that mirror the recent CRCM evolution (within version 4) and include some minor modifications in parameters associated with surface processes and O_3_ data. RCMs use GCMs to produce climate projections at a higher resolution on a regional (usually continental) scale (see Christensen et al. 2007 and references therein). CRCM simulations over two domains were considered: a domain covering North America (200 × 192 grid points) and a domain centered over Quebec (111 × 87 grid points), both with a horizontal grid-size mesh of 45 km (true at 60°N). Grids representing the RCM models are shown in Supplemental Material, Figure S2.

There are different sources of uncertainty that affect temperature projections (de Elía and Côté 2010; Déqué et al. 2007). These include *a*) the effects introduced by the RCM downscaling on temperature projections (here termed resolution); *b*) the GHG emission scenarios (SRES) based on different states of future human activities; *c*) the climate model (GCM) itself (each model is conceived with a different structural design, numerical scheme, and physical parameterizations); *d*) the initial conditions, which—because of the chaotic nature of the climate system—generate an intrinsic “natural variability” in the climate model response; and *e*) the domain (the area covered by the RCM simulation). In the present study, we assessed the influence of different simulations on the variability of temperature projections and thus on the variability of future mortality distributions.

To ensure that the 32 simulations covered most of the range of possible future temperature (minimum, maximum, and mean) simulations, we computed the differences between the average of future (2046–2065) and historical simulated temperature distributions (1971–2000) for the three temperature metrics (daily mean, minimum, and maximum temperature) based on the 32 simulations versus all simulations provided by the Ouranos Consortium on Regional Climatology and Adaptation to Climate Change (http://www.ouranos.ca/) (*n* = 127 simulations for mean temperatures, *n* = 111 for maximum and minimum temperatures). This analysis confirmed that the 32 simulations included in the present analysis covered most of the temperature variability distribution from all simulations available (see Supplemental Material, Figure S3).

Correction for simulated temperatures. GCMs and RCMs project future temperatures at specific locations with errors related to the scale of the model predictions and other factors (Mpelasoka and Chiew 2009). Correction methods originally developed for hydroclimatology studies (Teng et al. 2011) include notably constant scaling, daily scaling, and daily translation (DT) methods (Mpelasoka and Chiew 2009). For the present analysis, we used the DT method to derive correction factors based on differences between observed historical data for 1990–2007 from a local meteorological station and simulated data for the same location and time period, thereby accounting for errors related to scaling as well as other sources of error.

*Deaths attributable to historical and future temperatures*. Relationships between observed historical temperatures and mortality. We used separate generalized linear Poisson models to estimate associations between daily death counts [relative risks (RRs) compared with the daily mean death count over the entire period] and daily mean, maximum, and minimum observed temperatures during June, July, and August of 1990–2007 (Armstrong 2006). We used cubic B-splines of time with 5 degrees of freedom (splines package in R; http://www.R-project.org/) to control for secular trends in the mortality series (Hajat et al. 2007) and modeled the day of the season (1–92) using a spline with three knots (10th, 50th, and 90th percentiles) to control for seasonal patterns. To account for the nonlinear relationship between mortality and temperature, we modeled each temperature variable as a cubic spline with 5 knots (corresponding to 0, 25th, 50th, 75th, and 100th percentiles). Thus, a different RR was estimated for each degree of temperature, creating a set of RRs based on observed historical data. We also included daily mean levels of O_3_ in the regression as a simple continuous variable. To ensure that no autocorrelation remained in the residuals, we visually inspected partial autocorrelation plots and used the white noise statistical test (null hypothesis not rejected at *p* > 0.05; Lobato and Velasco 2004). We also visually inspected the plots of modeled Pearson residuals against the predicted values to verify that there was no important overdispersion.

Generation of 95% CIs of the ANs. There is no standard analytical way to estimate the 95% CI bounds of the distribution of a set of RRs (i.e., when there is one RR per degree temperature due to the nonlinear relation). We thus estimated the statistical uncertainty of the ANs calculated using the observed historical temperatures with 1,000 bootstrap samples, from which we selected the 2.5% and the 97.5% of the ANs based on the observed data, and the corresponding sets of RRs that produced them (i.e., corresponding point estimates per degree temperature produced with the same parameters as the Poisson model developed with the historical data).

The bootstrap samples were developed from the observed daily data as follows. For each of the 1,000 samples, we drew with replacement 18 times from day ones, the day twos, and so on (bootstrap samples were stratified by day of “summer”). Thus each of the 1,000 samples contained 1,656 days (92 days × 18 years).

Calculation of deaths attributable to temperatures. We first calculated the attributable fraction of daily deaths (*AF*) for each daily temperature metric value *T_i_* (mean, maximum, or minimum) using the three sets of RRs described above (i.e., one based on the observed historical data, and two based on individual bootstrap samples). We used Equation 1 to calculate the *AF* only when the RR was > 1 and the daily minimum, mean, and maximum temperatures were above 15°C, 20°C, and 20°C, respectively.

*AF*(*T_i_*) = [*RR*(*T_i_*) – 1]/*RR*(*T_i_*). [1]

The total number of ANs per year for the 1990–2007 and 2020–2037 periods for a given temperature metric (mean, maximum, or minimum) was then estimated using Equation 2:

*AN* = Σ[*AF*(*T_i_*) × *MDC* × *ND*(*T_i_*)], [2]

where *MDC* is the mean observed daily death count for the period 1990–2007 and *ND*(*T_i_*) is the number of days with the temperature metric (observed or simulated) of *T_i_*; values are summed from the minimum value of *T_i_* (i.e., 15°C, 20°C, and 20°C for daily minimum, mean, and maximum temperatures, respectively) for which the estimated RR for the temperature metric and mortality based on historical data was > 1 up to the maximum value of *T_i_*.

In our calculation of deaths attributable to future temperatures, we assumed that there would be no change in the mean daily death count in the future, no demographical change, no change in O_3_ levels, and no adaptation to heat from populations.

*Variability analysis*. We studied the influence of the different simulations on the future temperature and mortality distributions (2020–2037) focusing on daily mean temperature (not on daily maximum or minimum).

Temperature projection variability. We performed an ANCOVA in which the variable to be explained was the simulated daily mean temperature and the predictors were the simulation (*n* = 32) and the year modeled (*n* = 18).

Variability in ANs. We also used an ANCOVA to evaluate the influence of the set of RRs used to represent the temperature–mortality association, the simulation used to project future temperatures, and the year of the simulation on the estimated number of deaths attributable to temperature during each future year of 2020–2037. The three sets of RRs used included the set based on the observed historical data and the two sets of RRs based on the bootstrap data samples that generated the 95% CIs of ANs.

## Results

*Observations*. For the 18-year period 1990–2007 in Montreal, 61,356 nonaccidental deaths occurred during June–August, of which 79.9% were among people > 65 years of age. The average observed values for daily mean, maximum, and minimum temperatures were 20.4°C, 24.9°C, and 15.6°C for June, July, and August of 1990–2007. For the distributions of observed daily temperatures and O_3_ levels for the summers (i.e., June–August) of 1990–2007, see Supplemental Material, Table S1. The average daily O_3_ concentration for the same period was 25.3 μg/m^3^ (range, 0.83–76.1 μg/m^3^) (see Supplemental Material, Table S1).

*Simulated temperatures*. Historical temperatures. Average values of simulated daily mean temperatures for 1990–2007 over the 32 simulations were lower than observed values before the DT correction was applied (16.6°C compared with 20.4°C), whereas simulated mean daily temperatures were closer to the observed values after correction (e.g., mean 20.1°C) (Table 2). We found similar results with daily maximum and minimum temperatures (data not shown).

**Table 2:** Distribution of observed (1990–2007), historical simulated (1990–2007), and future simulated (2020–2037) average daily mean temperatures (°C) (*n *= 1,656 days).

Time period, quantile	Observed	Simulated [average (range)]	
		Uncorrected^*a*^	DT-corrected^*a*^
1990–2007
Minimum	9.6	4.55 (3.96–8.08)	7.62 (5.75–9.23)
1%	12.5	7.41 (6.94–7.99)	11.46 (10.58–12.36)
5%	15.0	9.80 (8.94–10.22)	14.02 (13.61–14.66)
25%	18.3	13.57 (13.17–13.89)	17.68 (17.33–18.03)
50%	20.5	16.56 (16.01–17.03)	20.22 (19.91–20.54)
75%	22.7	19.32 (18.79–20.06)	22.59 (22.42–22.89)
95%	24.5	23.71 (23.10–24.13)	25.62 (25.04–26.13)
99%	27.4	26.27 (25.44–27.82)	27.72 (26.84–28.83)
Maximum	29.2	28.77 (27.74–30.09)	31.37 (28.81–37.29)
Average	20.4	16.57 (16.02–16.97)	20.06 (19.85–20.34)
SD	3.2	4.21 (4.08–4.36)	3.56 (3.33–3.76)
2020–2037
Minimum	NA	5.50 (3.78–8.65)	8.15 (5.24–10.48)
1%	NA	8.43 (8.08–8.86)	12.07 (11.31–12.92)
5%	NA	10.55 (10.16–11.02)	14.69 (13.91–15.38)
25%	NA	14.38 (13.78–14.90)	18.47 (17.92–18.92)
50%	NA	17.68 (17.12–18.09)	21.10 (20.30–21.59)
75%	NA	20.70 (20.14–21.05)	23.57 (22.74–24.22)
95%	NA	24.80 (23.87–25.44)	26.58 (25.48–27.18)
99%	NA	29.27 (28.32–30.65)	28.76 (27.37–29.89)
Maximum	NA	29.71 (28.12–32.18)	32.21 (29.39–35.75)
Average	NA	17.62 (17.27–17.89)	20.94 (20.31–21.32)
SD	NA	4.33 (4.25–4.39)	3.66 (3.39–3.89)
NA, not applicable. ^***a***^Average (range) of the 32 simulations.			

Future temperatures. All DT-corrected simulations for June–August 2020–2037 suggested an increase in daily mean temperatures in Montreal compared with observed temperatures (Table 2): The average daily mean temperature from the 32 future simulations was 20.9°C (range: 20.3–21.3°C), compared with 20.4°C for the observed temperatures for June–August 1990–2007. We observed notable differences between the 32 DT-corrected and uncorrected values (uncorrected average daily mean, 17.6°C). However, the effect of the correction was not constant at all percentiles; for the future simulations, we observed greater differences in the ranges at higher and lower percentiles. For example, the average (range) of the corrected and uncorrected future simulated daily mean temperatures for the lowest percentile were 8.2°C (5.2–10.5°C) and 5.5°C (3.8–8.7°C), respectively, and for the 50th percentile were 21.1°C (20.3–21.6°C) and 17.7°C (17.1–18.1°C), respectively. Results were similar for daily maximum and minimum temperatures (data not shown).

Variability in temperature projections. Table 3 presents the percent of variance in daily temperature projections explained by the simulation and the year. Less than one percent of the daily temperature variation was explained by the choice of simulation model and year simulated.

**Table 3:** Effect of simulation and year on daily mean temperature projections from an ANCOVA model (*n* = 52,992).

Variable	Partial sum of squares	df	Mean squares	*F*	*p*-Value	η^2^
Year (*n *= 18; 2020–2037)	1,476	1	1,476	110.4	< 0.001	0.2%
Simulation (*n *= 32)	3,269	31	105	7.9	< 0.001	0.5%
Residuals	708,380	52,991	13	—	—	—
Abbreviations, df, degrees of freedom; η^2^, variance explained by the variable.						

*Deaths attributable to historical and future temperatures*. For the three sets of RRs for each temperature unit used to calculate deaths attributable to temperature, see Supplemental Material, Table S2. Applying the RR (> 1) of the temperature–mortality relationships, we estimated a mean of 62 (95% CI: 32, 86) attributable deaths per summer (i.e., June–August) for the years 1990–2007. For maximum and minimum historical daily temperatures, we estimated 55 (95% CI: 32, 79) and 38 (95% CI: 9, 61) attributable deaths, respectively. Deaths attributable to temperatures and 95% CIs calculated by bootstrapping are presented in Supplemental Material, Table S3.

**Figure 1:**
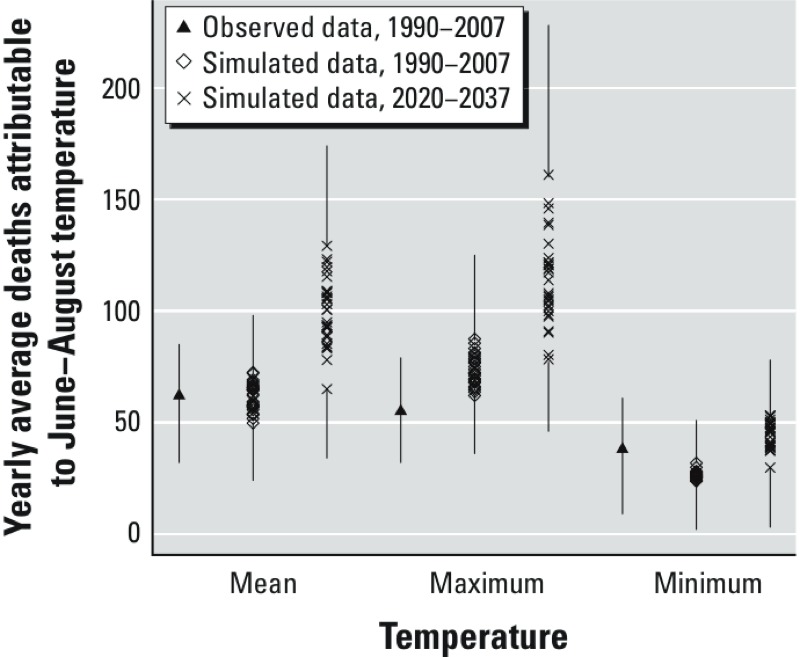
Estimated average annual deaths attributable to temperature (daily mean, daily maximum, or daily minimum) during June–August based on observed data for 1990–2007 and simulated data for 1990–2007 and 2020–2037. Simulated data are based on 32 simulations from RCMs and GCMs and corrected by the DT method. Deaths attributable to simulated temperatures were estimated with a set of RRs based on the observed historical data. The upper lines represent the ANs calculated with the set of RRs that generated the upper 95% CI bound of the ANs for the highest attributable number of deaths; the lower lines represent the ANs calculated with the set of RRs that generated the lower 95% CI bound of the ANs for the lowest attributable number of deaths. The three sets of RRs were also used to generate the attributable numbers for the observed data.

The estimated numbers of deaths attributable to daily mean, maximum, and minimum temperatures during June–August 2020–2037 based on the 32 DT-corrected simulations are presented in Figure 1, along with estimated numbers of deaths attributable to temperatures during 1990–2007 based on observed and DT-corrected simulated temperatures. Average numbers of deaths attributable to daily mean temperature during each year for 2020–2037 based on all 32 simulations were higher than the average number based on observed mean daily temperatures for 1990–2007. For maximum and minimum daily temperatures, 100% and 72% of the projections, respectively, produced estimated ANs of daily deaths above the average estimated for observed daily temperatures in 1990–2007. Average ANs of deaths based on simulated daily mean temperature during each year for 1990–2007 were similar to the average numbers based on observed mean daily temperatures for that time period. However, we observed differences in ANs between simulated minimum and maximum temperatures for this period.

Variability in mortality projections. Using the RR based on the observed historical data and the 32 simulations to estimate ANs for 2020–2037, we noted a high degree of variability in summer deaths attributable to daily temperatures (range, 65–129 summer deaths for mean daily temperatures; 78–161 for maximum daily temperatures; and 30–53 for minimum daily temperatures) (see Supplemental Material, Table S3).

Table 4 presents the percentage of variance in future yearly deaths attributable to temperature projections that is explained by the simulation, the year, and the set of RRs used to calculate the ANs. A higher part of the variability in mortality projections than in temperature projections was explained by the simulation: Six percent of the yearly variation of deaths attributable to future temperatures was explained by the choice of the simulation, compared with < 1% for the projections of temperatures. Nonetheless, most of the variability in mortality projections (38%) was related to the temperature–mortality RRs used to estimate the attributable fraction of heat-related deaths.

**Table 4:** Effect of simulation, year, and set of RRs on deaths attributable to future temperatures by season from an ANCOVA model (*n* = 1,728).

Variable	Partial sum of squares	df	Mean squares	*F*	*p*-Value	η^2^
Set of RRs (*n *= 3)	2,143,137	2	1,071,568	607.3	< 0.001	38.0%
Year (*n *= 18; 2020–2037)	163,979	1	163,979	92.9	< 0.001	2.9%
Simulation (*n *= 32)	342,493	31	11,048	6.3	< 0.001	6.1%
Residuals	2,987,066	1,693	1,764	—	—	—
Abbreviations, df, degrees of freedom; η^2^, variance explained by the variable.						

**Figure 2:**
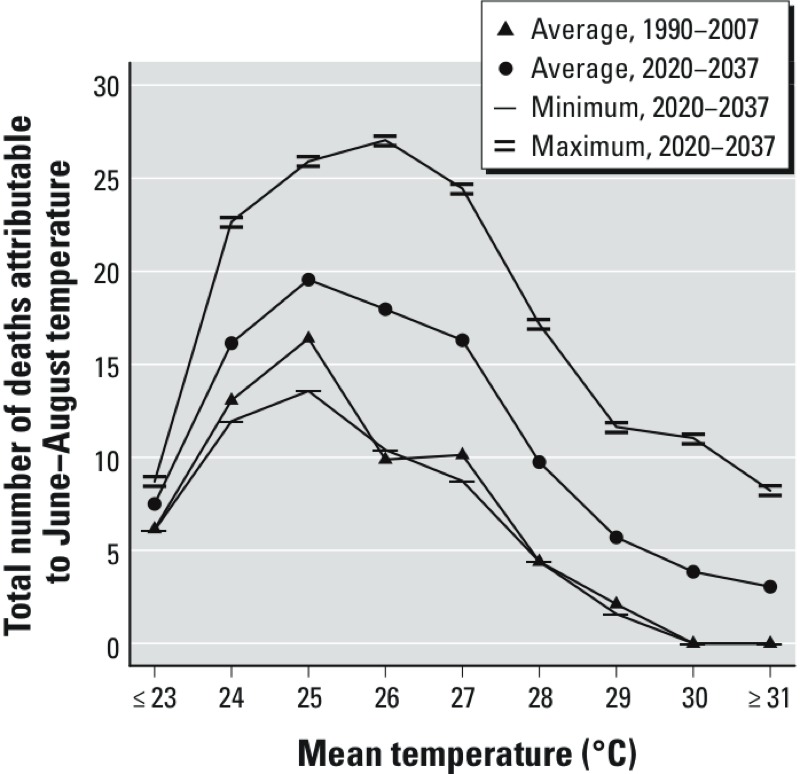
Yearly estimates of average, minimum, and maximum numbers of deaths during June–August attributable to observed temperatures in 1990–2007 and predicted temperatures in 2020–2037 based on 32 simulations (corrected using the DT method) according to mean daily temperature (°C).

Figure 2 shows the yearly average estimated number of attributable deaths associated with each daily mean temperature value with RR > 1; the numbers for each distribution sum to the average total estimated number of attributable deaths. Most of the estimated attributable deaths occur on days with daily mean temperatures between 24°C and 28°C; this reflects the number of days with temperatures in this range.

## Discussion

In the present study, we estimated the variability in projections of deaths attributable to temperatures in Montreal during June–August 2020–2037 using 32 RCM and GCM temperature simulations (with different climate models, SRES, domains, versions, and members). Using DT-corrected simulated June–August temperatures, we found an increase in estimated numbers of deaths attributable to daily mean ambient temperatures during 2020–2037, with a large variability ranging from 34 to 174 deaths per summer, compared with 62 deaths attributable to daily observed mean temperatures in 1990–2007. We found that a small portion of the estimated variability in mortality projections was due to the different simulations (i.e., variability due to characteristics of the simulations); most of the variability was associated with the RR used to calculate deaths attributable to temperature.

The uncertainty related to the temperature–mortality relationship had much more impact on heat-related mortality projections than did uncertainty due to climate models. This may be a result of the chosen temperature simulations. Although the simulations chosen covered an important part of the temperature variability from all simulations available, we did not include extreme simulations in our study (see Supplemental Material, Figure S3). Our choice of simulations may thus contribute to underestimating the contribution of the climate model projections. Furthermore, the uncertainty related to the temperature–mortality relationship may be large because of the propagation of the errors associated with the repeated use of the same mortality risk for a given temperature that occurs frequently; small changes in the risk function are magnified when applied repeatedly over numerous summer days. Future work is thus needed to better assess the uncertainty of the temperature–mortality relationship and climate model projections. Finally, we used a crude way to apportion the variation in the future ANs to different sources of variations, using only the sets of RRs that generated the lower and upper bounds of the 95% CI of the ANs and not the shape of the distribution of probable RRs. The explained variance by the sets of RRs thus represents an upper estimate.

Uncorrected simulated values of historical temperatures were quite different from the observed data. Therefore, to examine the variability in mortality projections, we corrected the simulated temperatures using the DT method. We used this method because it allows the validation of historical simulations on the basis of observed temperatures. Future work should seek to validate and improve different methods of correcting simulated temperatures in mortality projections, as is done in hydroclimatology studies (Teng et al. 2011).

To date, studies published on the near-future impact of summer temperatures on mortality in Montreal have reported an increase in deaths attributable to heat relative to current numbers of deaths in the summer (Cheng et al. 2008; Doyon et al. 2008; Martin et al. 2011). Although these studies used different methods to estimate future mortality, it is still possible to compare their results with our ours. Martin et al. (2011) calculated a predicted change in annual heat-related mortality rate per 100,000 population, and they estimated an increase of 152 heat-related attributable deaths per summer for the period 2031–2050, compared with 1981–2000. Doyon et al. (2008) calculated the equivalent of 79 summer heat-related deaths for 2020 (2% increase) and 81 deaths, for 2050 (6% increase). Cheng et al. (2008) estimated 96.3 summer heat-related deaths per year during 2040–2059. These results correspond to the range of our results. Regarding our results on factors affecting the variability in mortality projections, some differences are likely due to the RRs used to estimate the ANs of heat-related deaths. Lower numbers of summer heat-related deaths are likely in southern countries because heat thresholds are generally higher in communities closer to the equator (Hajat and Kosatsky 2010).

Methods for estimating mortality projections inevitably rely on assumptions concerning heat-related mortality relationships. In the present study, we chose to not take into account lag effects (Goldberg et al. 2011), harvesting, or mortality displacement (Zanobetti et al. 2002), nor did we consider intraurban variations of temperatures and risks (Smargiassi et al. 2009). Future studies should consider these aspects. We estimated only nonaccidental causes of death, and we did not conduct specific analyses for cardiovascular or respiratory causes of death (Goldberg et al. 2011; Halonen et al. 2011), even though the distribution of specific causes of death may vary in the future. Thus, further work should address these aspects. Our study also had other limitations: We assumed the same mean daily death count in the future, no demographic changes, and no population adaptation to heat, such as through access to air conditioning (Rogot et al. 1992). It is difficult to conclude what the impacts of these assumptions might be. On one hand, with demographic changes there are likely to be more vulnerable populations (elderly populations in particular); on the other hand, adaptations and mitigation measures may reduce climate impacts (Patz et al. 2008). New climate change impact studies taking into account these specific adaptation and mitigation measures should be performed. Humidity or dew point simulation for future years are needed to address the effect of these other climate factors.

Effects of climate change on health will affect most populations in the next decades, and put the lives and well-being of billions of people at increased risk. Our results suggest that the choice of the RR estimate for the association between temperature and mortality may be important in reducing uncertainty in mortality projections.

## Supplemental Material

(1.2 MB) PDF
