# Supplementary material for: Variability in Temperature-Related Mortality Projections under Climate Change
Source: Environ Health Perspect. 2014 Jul 18;122(12):1293–8. doi: 10.1289/ehp.1306954 (PMC4256694; doi:10.1289/ehp.1306954)
Supplement: (1.2 MB) PDF [file ehp.1306954.s001.508.pdf]

Environmental Health Perspectives doi:10.1289/ehp.1306954

Variability in Temperature-Related Mortality Projections under Climate Change

Tarik Benmarhnia, Marie-France Sottile, Céline Plante, Allan Brand, Barbara Casati, Michel Fournier, and Audrey Smargiassi

Table of Contents **Page**

Figure S1. GCM models grid. NB: the lines represent the different GCM used (see Table 2) 2

Figure S2. RCM models grid 3

Figure S3. Comparison between the 32 temperatures simulations used with 127 simulations available at Ouranos Consortium 4

Table 1S. Observed temperatures (T°), relative humidity, ozone levels for the period 1990-2007 (n=1656 days) 5

Table S2. RR from the three sets for each temperature unit (in °C) in Montreal, for daily mean, maximum and minimum temperatures 6

Table S3. Estimated annual number of deaths attributable to observed mean, minimum, or maximum daily temperature of June–August 1990-2007, and minimum, and maximum numbers of annual deaths attributable to projected temperature data for 2020–2037 from 32 simulations 8

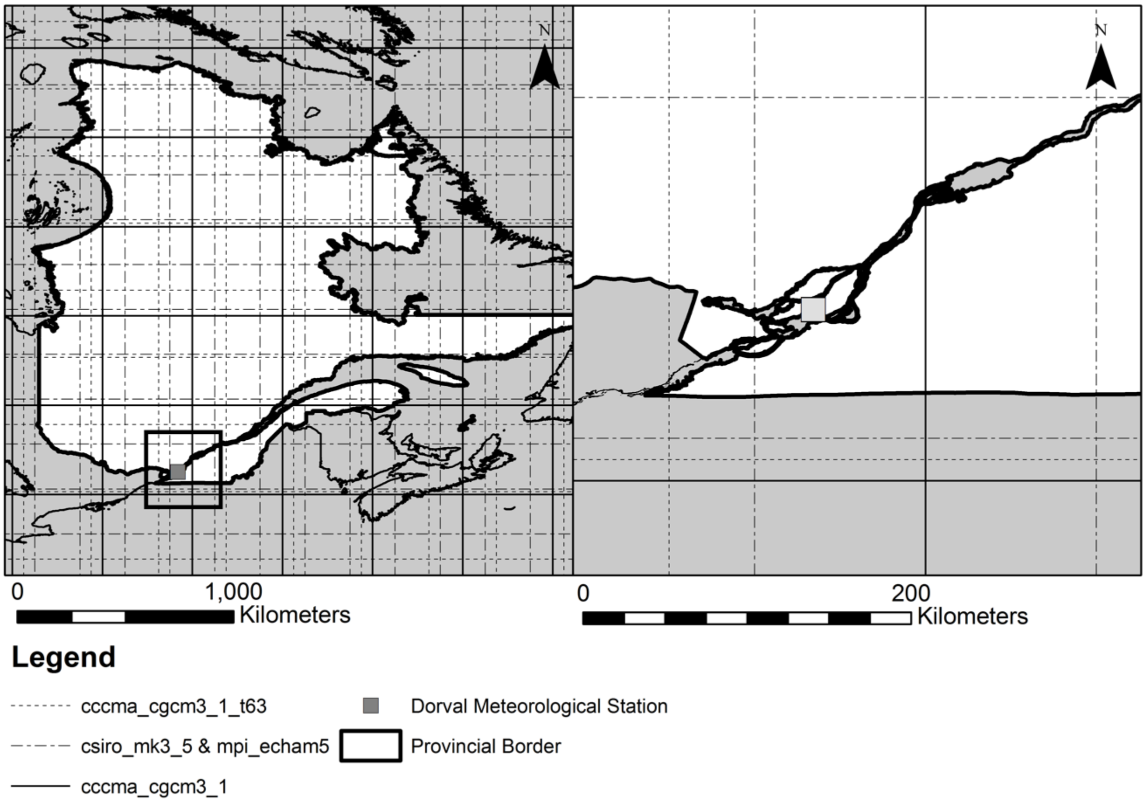

Figure S1. GCM models grid. NB: the lines represent the different GCM used (see Table 2). The area shown represents the Province of Quebec (left) and the city of Montreal (right).

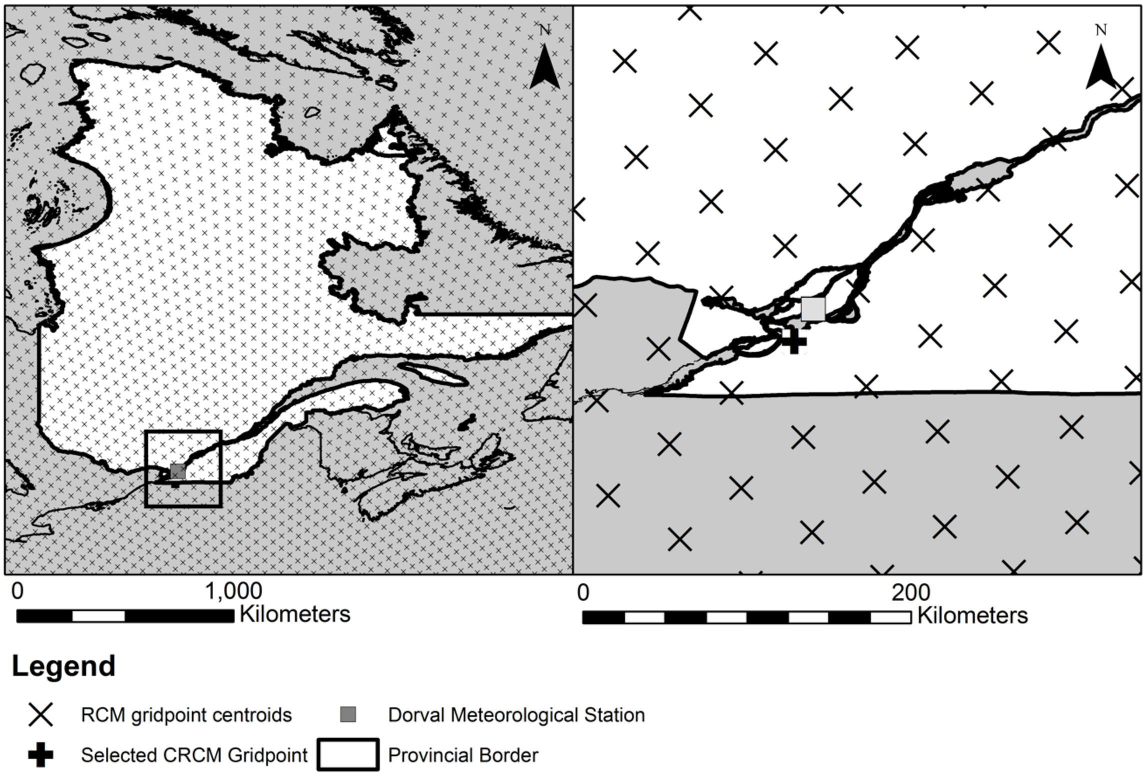

Figure S2. RCM models grid. The area shown represents the Province of Quebec (left) and the city of Montreal (right).

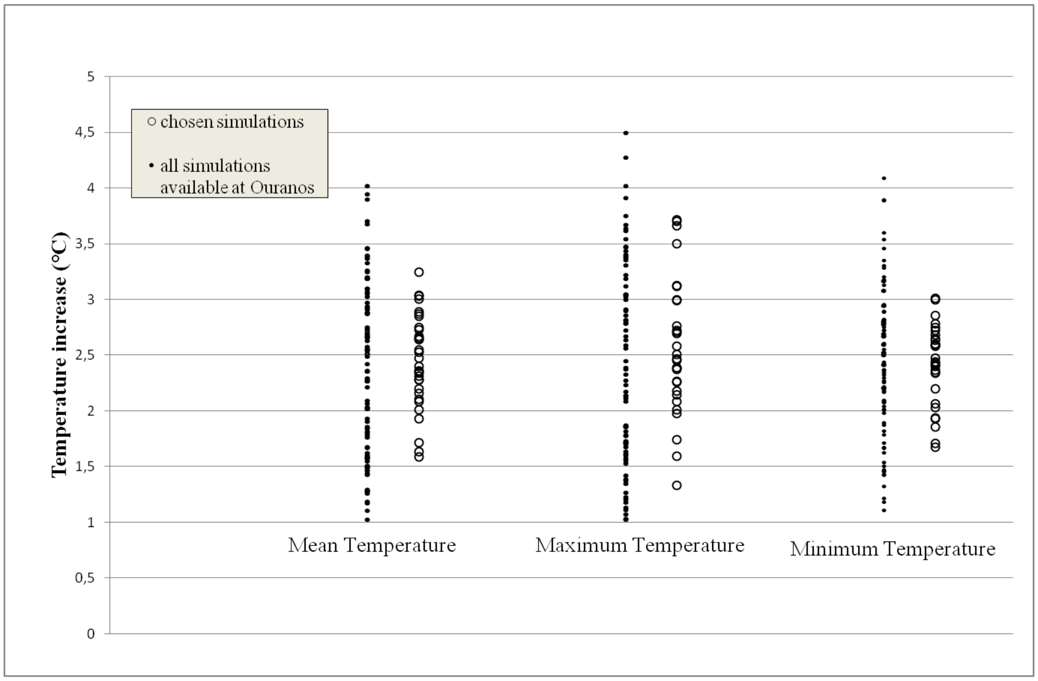

Figure S3. Comparison between the 32 temperatures simulations used with 127 simulations available at Ouranos Consortium. Differences between the mean of the future (2046-2065) and of the historical simulated temperature distributions (1971-2000) (deltas of the mean of the three temperature variables, i.e. daily mean. minimum and maximum temperatures) for the simulations chosen and for all available simulations at the Ouranos consortium on climate change (n=127 simulations for mean temperatures, n=111 for maximum and minimum temperatures).

Table 1S. Observed temperatures (T°), relative humidity, ozone levels for the period 1990-2007 (n=1656 days).

**Quantile**	**Mean T° (C°)**	**Maximum T° (C°)**	**Minimum T° (C°)**	**O_3 (µg/m^3)**
Minimum	9.6	12.0	3.1	0.83
1%	12.5	15.4	7.0	5.4
5%	15.0	18.6	9.5	9.8
25%	18.3	22.5	13.3	17.1
50%	20.5	25.0	15.8	23.5
75%	22.7	27.5	18.2	31.3
95%	24.5	30.7	21.2	47.7
99%	27.4	32.5	23.1	
Maximum	29.2	35.4	25.8	76.1
Mean	20.4	24.9	15.6	25.3
Standard deviation	3.24	3.70	3.57	1

Table S2. RR from the three sets for each temperature unit (in °C) in Montreal, for daily mean, maximum and minimum temperatures.

Temperature	Mean °C RR, observed historical data	Mean °C RR, 2.5% of bootstrap estimates	Mean °C RR, 97.5% of bootstrap estimates	Max. °C RR, observed historical data	Max. °C RR, 2.5% of bootstrap estimates	Max. °C RR, 97.5% of bootstrap estimates	Min. °C RR, observed historical data	Min. °C RR, 2.5% of bootstrap estimates	Min. °C RR, 97.5% of bootstrap estimates
5	0.95	0.85	0.95	NA	NA	NA	0.93	0.91	0.83
6	0.95	0.85	0.95	NA	NA	NA	0.93	0.91	0.86
7	0.95	0.86	0.94	NA	NA	NA	0.94	0.92	0.88
8	0.95	0.87	0.94	NA	NA	NA	0.94	0.93	0.90
9	0.95	0.87	0.93	1.01	1.02	1.12	0.94	0.93	0.92
10	0.94	0.88	0.92	1.00	1.02	1.10	0.95	0.94	0.94
11	0.94	0.89	0.92	0.99	1.01	1.09	0.96	0.95	0.95
12	0.94	0.90	0.92	0.99	1.00	1.07	0.96	0.96	0.96
13	0.94	0.90	0.91	0.98	0.99	1.05	0.97	0.97	0.97
14	0.95	0.91	0.91	0.97	0.99	1.04	0.98	0.98	0.98
15	0.95	0.92	0.91	0.97	0.98	1.02	0.99	0.99	0.98
16	0.95	0.93	0.92	0.96	0.97	1.01	0.99	0.99	0.99
17	0.95	0.94	0.93	0.96	0.97	1.00	1.00	0.99	1.00
18	0.96	0.95	0.95	0.96	0.96	0.99	1.01	0.99	1.02
19	0.97	0.96	0.97	0.96	0.96	0.98	1.02	1.00	1.04
20	0.98	0.98	0.99	0.96	0.96	0.97	1.04	1.01	1.07
21	0.99	0.98	1.01	0.96	0.96	0.97	1.06	1.03	1.09
22	1.00	0.98	1.01	0.96	0.96	0.97	1.09	1.05	1.12
23	1.02	1.00	1.03	0.97	0.97	0.97	1.12	1.08	1.16
24	1.05	1.02	1.07	0.98	0.97	0.97	1.16	1.11	1.19
25	1.09	1.06	1.12	0.98	0.98	0.98	1.20	1.14	1.23
26	1.14	1.12	1.19	0.99	0.99	0.99	1.24	1.17	1.26
27	1.20	1.18	1.28	1.00	1.00	1.00	1.28	1.21	1.30
28	1.27	1.25	1.38	1.02	1.01	1.03	1.32	1.24	1.34
29	1.35	1.33	1.50	1.05	1.03	1.07	1.37	1.28	1.38
30	1.42	1.42	1.63	1.08	1.05	1.12	NA	NA	NA
31	1.51	1.51	1.76	1.12	1.07	1.19	NA	NA	NA
32	1.60	1.60	1.91	1.17	1.09	1.27	NA	NA	NA

Temperature	Mean °C RR, observed historical data	Mean °C RR, 2.5% of bootstrap estimates	Mean °C RR, 97.5% of bootstrap estimates	Max. °C RR, observed historical data	Max. °C RR, 2.5% of bootstrap estimates	Max. °C RR, 97.5% of bootstrap estimates	Min. °C RR, observed historical data	Min. °C RR, 2.5% of bootstrap estimates	Min. °C RR, 97.5% of bootstrap estimates
33	1.69	1.70	2.08	1.23	1.12	1.36	NA	NA	NA
34	1.79	1.81	2.25	1.29	1.14	1.46	NA	NA	NA
35	1.89	1.93	2.44	1.35	1.17	1.57	NA	NA	NA
36	2.01	2.05	2.65	1.42	1.20	1.70	NA	NA	NA
37	2.12	2.18	2.88	1.50	1.23	1.83	NA	NA	NA
38	NA	NA	NA	1.58	1.26	1.97	NA	NA	NA
39	NA	NA	NA	1.66	1.29	2.13	NA	NA	NA
40	NA	NA	NA	1.74	1.32	2.29	NA	NA	NA
41	NA	NA	NA	1.83	1.35	2.47	NA	NA	NA
42	NA	NA	NA	1.93	1.38	2.67	NA	NA	NA
43	NA	NA	NA	2.03	1.42	2.88	NA	NA	NA

NA: Not applicable

Table S3. Estimated annual number of deaths (with 95% CI[a]) attributable to observed mean, minimum, or maximum daily temperature of June–August 1990-2007, and minimum, and maximum numbers of annual deaths attributable to projected temperature data for 2020–2037 from 32 simulations.

Daily temperature metric	AN based on observed temperatures (1990-2007)	Minimum AN based on simulated temperature (2020-2037)	Maximum AN based on simulated temperature (2020-2037)
Mean daily temperature	62 (32, 86)	65 (34, 89)	129 (67, 174)
Maximum daily temperature	55 (32, 79)	78 (46, 109)	161 (95, 228)
Minimum daily temperature	38 (9, 61)	30 (3, 46)	53 (15, 78)

[a]CI bounds for estimates based on simulated data represent the 2.5th and 97.5th percentiles of 1,000 bootstrap samples.
